# Arthroscopic biceps tenodesis using press-fit bony plug: a case series study

**DOI:** 10.1007/s00264-023-06021-3

**Published:** 2023-11-04

**Authors:** Ahmed Mahmoud Gad, Sherif Hamdy Zawam

**Affiliations:** Department of Trauma and Orthopedics, Faculty of Medicine, Cairo University, Giza, Egypt

**Keywords:** Biceps, Tenodesis, Press-fit, Shoulder arthroscopy

## Abstract

**Purpose:**

To assess the feasibility, operative time, clinical outcomes, possible complications, and failure rates of all-through arthroscopic biceps tenodesis using press-fit bony plug technique.

**Methods:**

This prospective case series study involved 30 skeletally mature patients with long head of biceps pathology (tendinitis after failure of conservative treatment, subluxation, dislocation, or tendon tears). All patients were followed up for 24 months at least.

**Results:**

Twenty-nine patients regained full shoulder and elbow range of motion; one case suffered from reflex sympathetic dystrophy. There was a significant improvement in the constant, ASES, and VAS scores when comparing the pre-operative and post-operative values. The average biceps strength was 96% compared to the opposite healthy side. No cases were complicated by neuro-vascular deficits or failure of the tenodesis.

**Conclusion:**

Press-fit biceps tenodesis is safe and accessible with low economic demands. We recommend this technique to be used more often when addressing patients with long head of biceps pathologies.

**Registration data:**

Registration number: N-1562023.

Registration date: June 2022 “Retrospectively registered”.

## Introduction

Long head of biceps (LHB) lesions are among the most common causes of shoulder pain. The long intra-articular course makes the long head susceptible to various pathologies. The lesions vary from biceps tendinopathy, partial or complete tendon tears, subluxation, or even dislocation of the tendon [1–4].

It commonly occurs with other shoulder pathologies such as rotator cuff tears or superior labrum anterior to posterior lesions (SLAP) [3–6].

In mild cases of tendinopathy, conservative management in the form of medical treatment, physiotherapy, or corticosteroid injection is tried initially. However, in other cases, surgery is usually indicated [7].

Surgery is either tenotomy or tenodesis after resection of the affected part of the tendon. Although tenotomy is much easier to perform with less operative time, it is indicated in patients with old age or patients with low demand. This is because of the complications encountered such as cosmetic deformity or muscle cramps if performed in high-demand individuals [8–12].

Arthroscopic biceps tenodesis has been performed with several techniques such as bio-screws or suture anchors [13–16].

In our study, we performed all-through arthroscopic biceps tenodesis using the press-fit bony plug technique after resection of the diseased tendon segment. Our aim was to assess the feasibility, operative time, clinical outcomes, possible complications, and failure rates of press-fit biceps tenodesis.

## Patients and methods

Our prospective case series study was conducted in a tertiary hospital between the period of December 2019 and November 2022. Our inclusion criteria included skeletally mature patients (> 18 years) with long head of biceps pathology (tendinitis after failure of conservative treatment, subluxation, dislocation, or tendon tears). Biceps lesions may be isolated or associated with rotator cuff tears or SLAP lesions. The exclusion criteria were any patient with recurrent shoulder subluxation, dislocation, arthritic shoulder joint, or previous fractures around the shoulder. After obtaining the ethical committee approval, informed consent was signed by all patients participating in the study.

A detailed history was taken and physical examinations were done on all patients. Special tests were performed including Speeds, O'Brien’s, and Yergason’s tests and an MRI was ordered to confirm the diagnosis.

Surgery was performed under general anaesthesia in the beach chair position. Routine shoulder scope portals were made, whereas the posterior portal was used for visualization and the anterior shoulder portal was used as a working portal.

**Fig. 1:**
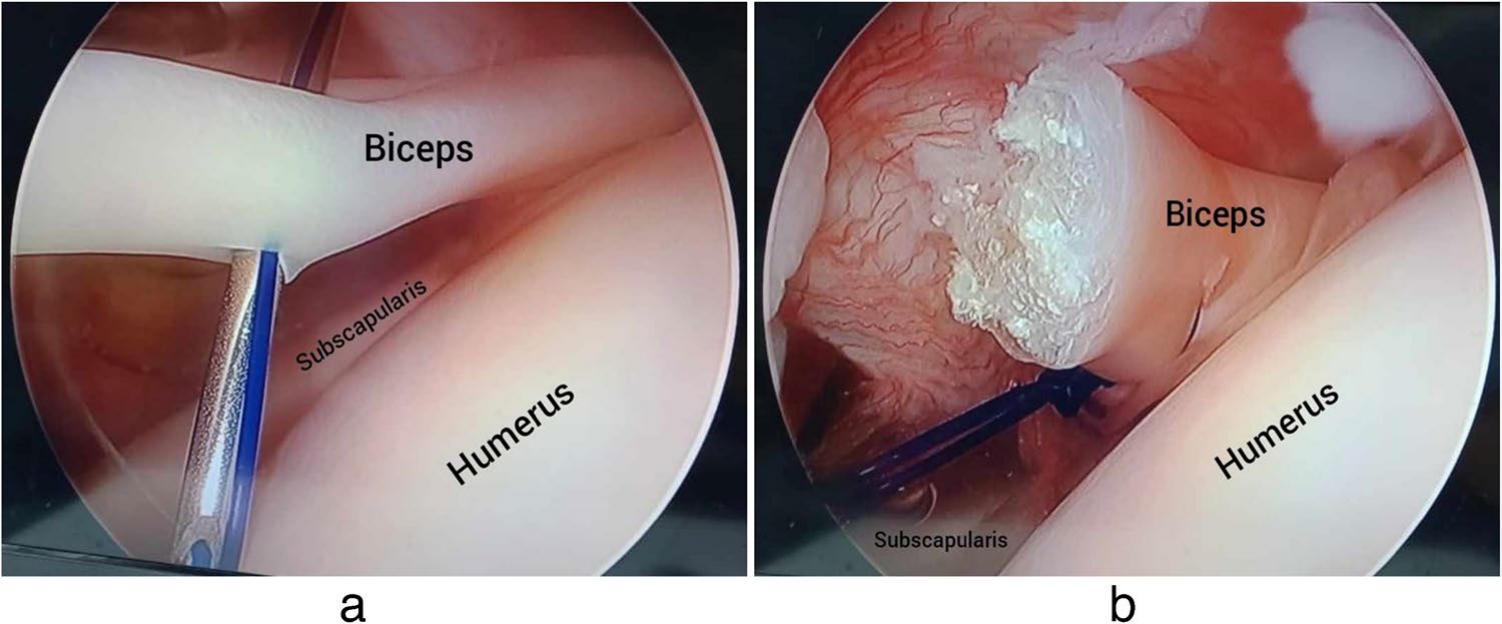
Introducing a spinal needle to pass Prolene/PDS sutures through the biceps tendon (intra-articular arthroscopic view of left shoulder from the posterior portal). **a** Before cutting the proximal end. **b** After cutting the proximal end

Through the anterior portal, a spinal needle was used to introduce Prolene or PDS sutures through the biceps tendon nearly 2 cm above the estimated point of later tunneling. These sutures will act as slings for the biceps tendon to avoid the distal escape of the tendon after the proximal cut from the glenoid. Also, they will be used to pass the tendon into the tunnel (Fig. 1).

**Fig. 2:**
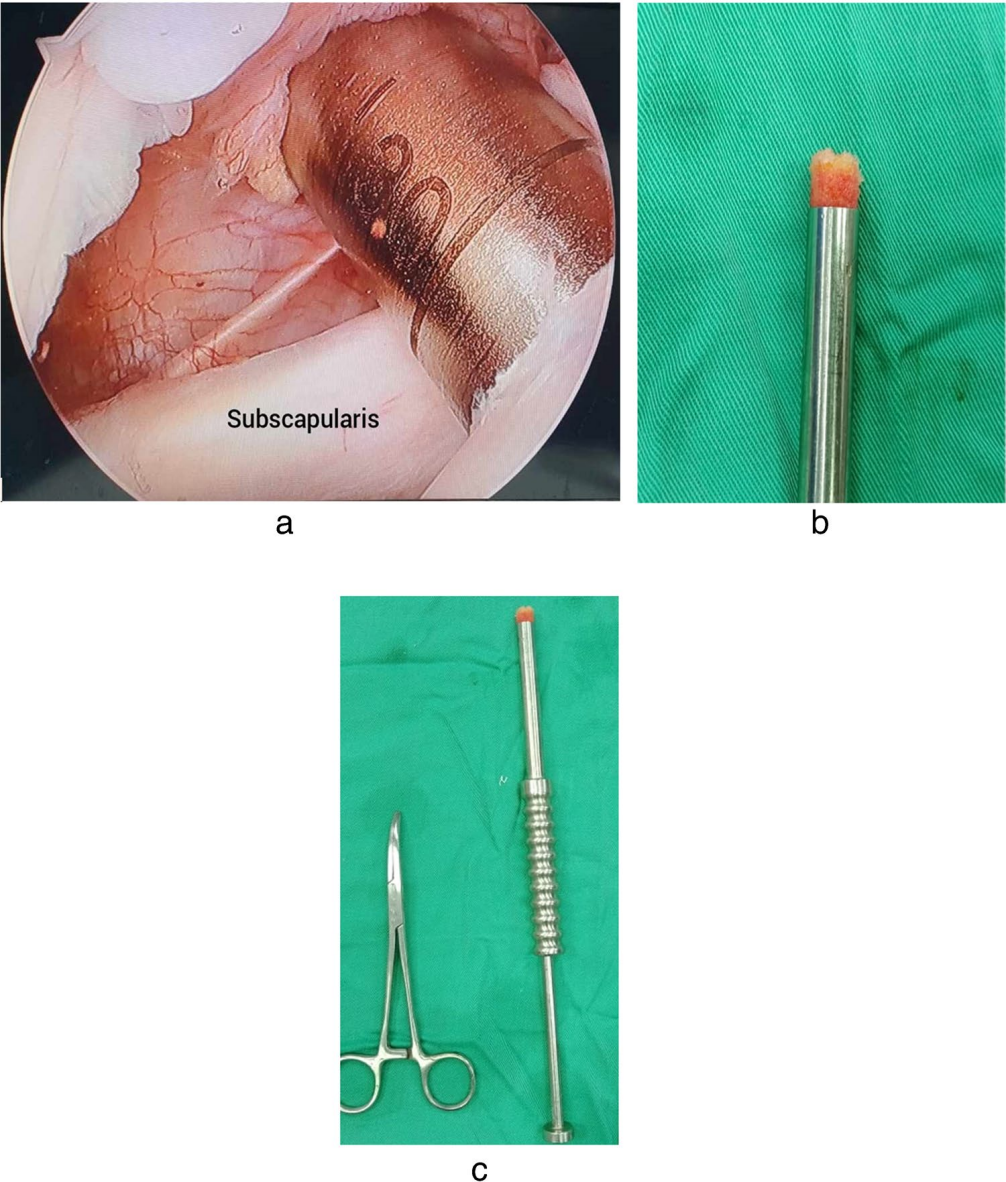
Bone plug extraction. **a** Intra-articular arthroscopic view of the left shoulder from the posterior portal. **b**, **c** After extraction of the bone plug

Using a trephine drill 8 mm in size, a tunnel hole with 2–2.5-cm depth was done in the bicipital groove just lateral to the subscapularis insertion. A 2–2.5-cm bone plug was extracted from the trephine drill to be used for later fixation (Fig. 2).

**Fig. 3:**
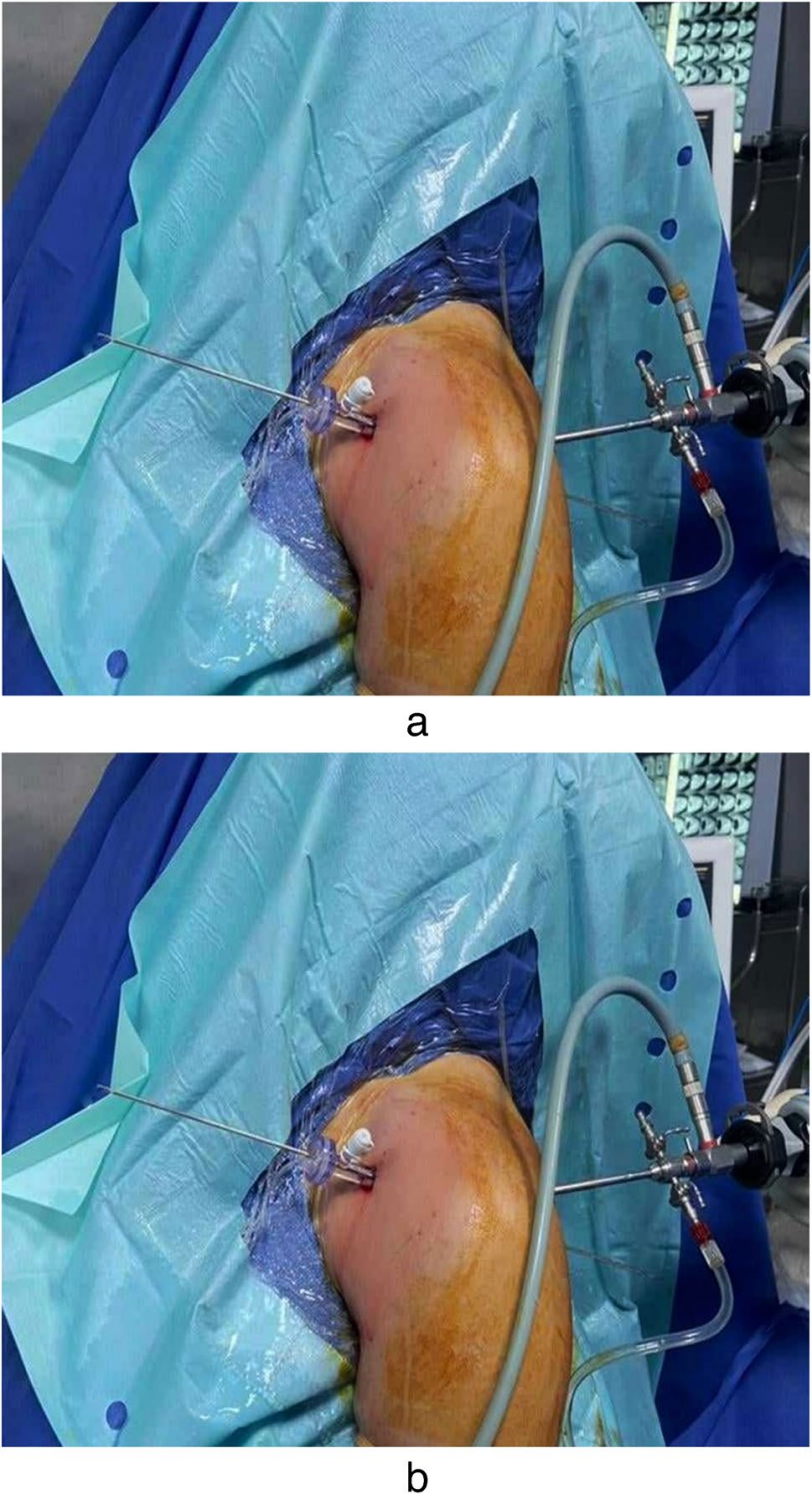
**a** Passing wire inserted through the depth of the hole from the anterior cannula and hammered through the posterior far cortex. **b** The free ends of the Prolene sutures shuttled using the passing wire through the hole and withdrawn from the posterior aspect of the arm to pull the biceps in the tunnel

The posterior cortex was preserved. A passing wire was inserted through the depth of the hole and hammered through the posterior far cortex. The free ends of the Prolene sutures were shuttled using the passing wire through the hole and withdrawn from the posterior aspect of the arm. Traction on the sutures would pass the proximal biceps stump into the tunnel (Fig. 3).

**Fig. 4:**
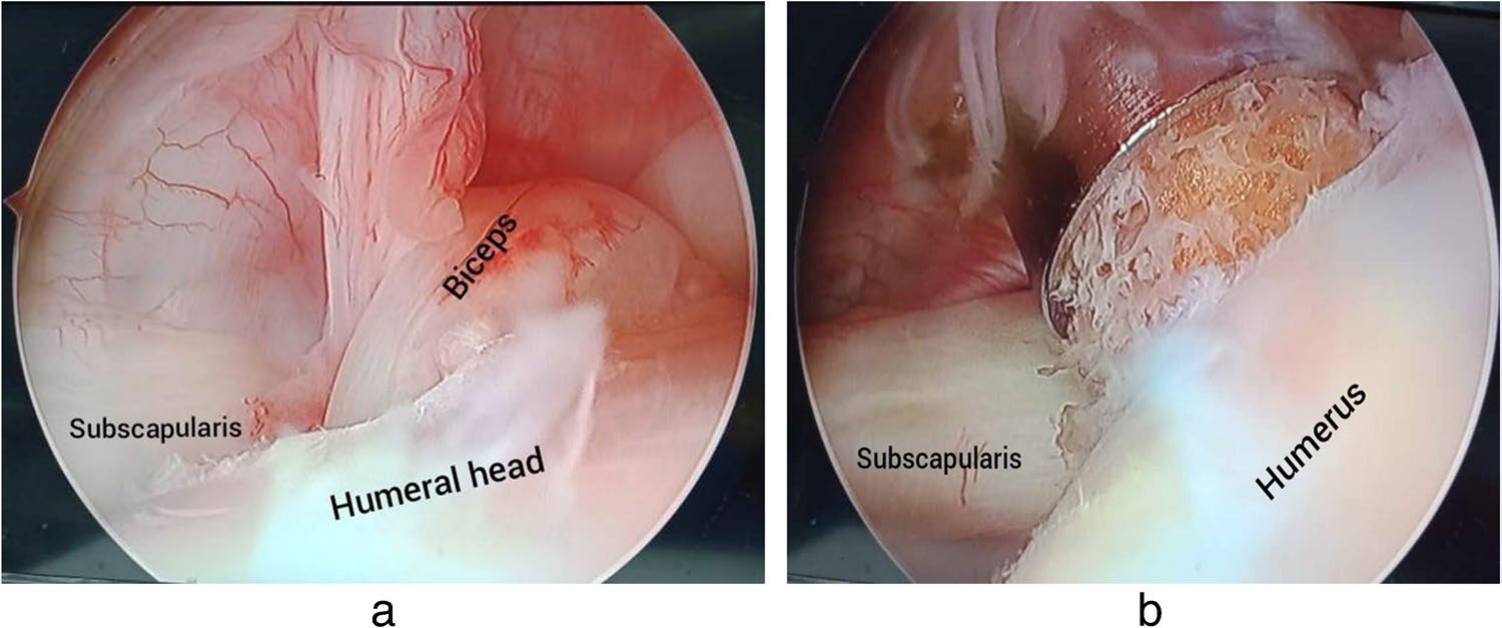
**a** Passing the biceps tendon into the tunnel. **b** Fixation by the press-fit bone plug

While maintaining the biceps stump in the hole, the extracted bone plug was inserted through the introducer at the rim of the hole and then hammered gradually until it fit in the tunnel (Fig. 4).

**Fig. 5:**
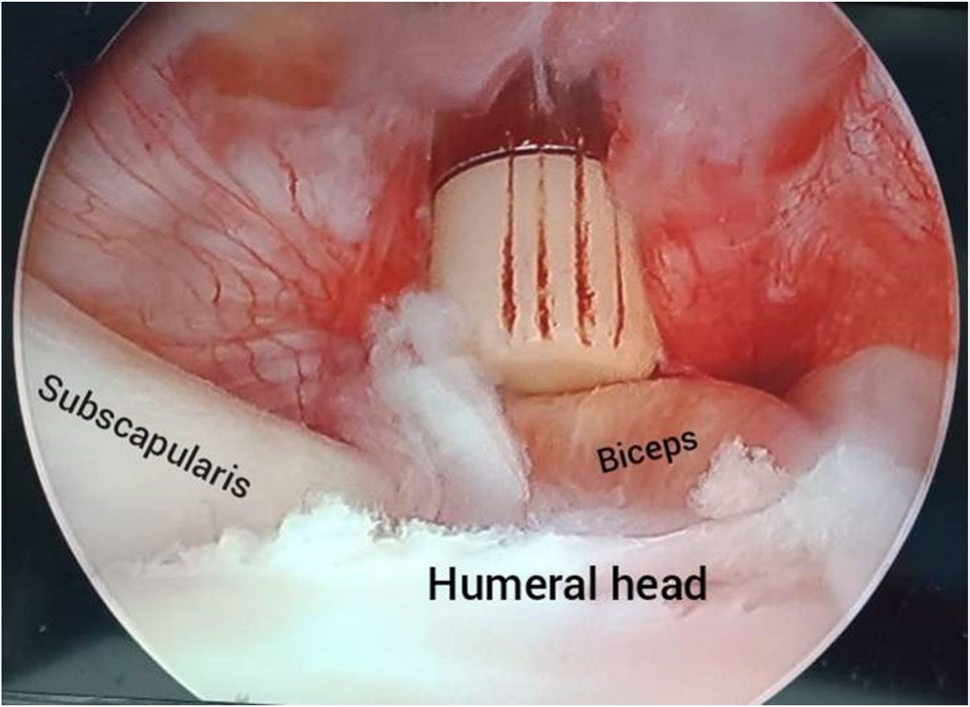
Final impaction of the bony plug

Final impaction of any elevated part of the plug (Fig. 5). Traction and probing of the tendon were done to ensure the stability of the tenodesed tendon after the plug insertion.

The patients were evaluated at three, six, nine, 12, and 24 months using the constant, VAS, and ASES scoring systems. Shoulder, elbow range of motion (ROM), and biceps strength were compared to the opposite side.

## Post-operative care

Elbow passive and active ROM were allowed in all cases immediately after surgery.

In cases of isolated biceps lesions, arm sling was intermittently used to control pain for two weeks. Shoulder passive and active ROM were initiated from the second day. Gradual strengthening exercises were started after six weeks.

In cases of associated rotator cuff repair, the patients were instructed to wear the arm sling for six weeks. Passive shoulder ROM was started after surgery. Pendular exercises were initiated from the 3rd post-operative week. Active exercises were allowed after six weeks and strengthening exercises were started after the months.

## Results

Thirty patients were included in our study, 18 males and 12 females with an age range from 27 to 54 years old and a mean age of 41 years old. Fifteen patients (50%) were smokers, seven cases (23.3%) were diabetics but controlled, and the dominant shoulder (right) was affected in 21 cases (70%). The injury types varied from isolated biceps tenosynovitis in six cases (20%), partial biceps tendon tear in five cases (16.7%), tendon subluxation associated with subscapularis injury in six cases (20%), and LHB injury associated with other rotator cuff tears in 13 cases (43.3%). After a detailed shoulder joint examination, the average constant, ASES, and VAS scores were calculated to be compared to post-operative results. The average operative time for the isolated biceps procedure was 22 min (range 17–29). The average length of hospital stay was 2.5 days. All patients were followed up for at least two years **(**Table 1).

**Table 1:** Patients’ demographics

Demographic data	
Age	Average: 41 years Range: 27–54 years
Sex	Male: 18 Female: 12
Side	Right: 21 Left: 9
Classification	Isolated biceps tenosynovitis: 6 cases (20%) Partial biceps tendon tear: 5 cases (16.7%) Tendon subluxation with sub-scapularis injury: 6 cases (20%) Biceps injury associated with other rotator cuff tears: 13 cases (43.3%)
Co-morbidities	Smokers: 15 (50%) Diabetes mellitus: 7 (23.3%)
Operative time	Average: 22 min Range: 17–29 min
Hospital stay	Average: 2.5 days Range: 2–4 days

Pre-operatively, the average constant score was 47 points (range 18–62) and showed significant improvement compared to 94 post-operatively (range 85–98) (*p* value < 0.05). Also, the ASES varied significantly from a pre-operative score of 43.3 points to 95 points at the last follow-up with *p* value = 0.01. When comparing the pre- versus post-operative VAS scores, we found that the scores dramatically improved from an average score of 6.5 pre-operatively to 0.6 post-operative (last follow-up) score (*p* value = 0.003).

Regarding the elbow and shoulder range of motion, all the patients regained the full range of the elbow and all except one patient regained the full shoulder range of motion. This patient suffered from reflex sympathetic dystrophy which was successfully managed by medical treatment and physiotherapy.

At three months, one patient (3.3%) reported pain in the bicipital groove, and three cases had mild tenderness on palpation. These symptoms and signs disappeared six months after surgery. The average biceps strength was 96% at the final follow-up when compared to the opposite healthy side. No cases were complicated by neuro-vascular deficits post-operative either transient or permanent. There were no cases who complained of cosmetic deformity (Popeye’s sign), biceps cramping, or failure of the tenodesis.

## Discussion

Long head of biceps pathologies are among the most common lesions of the shoulder joint. Most shoulder pathologies may result in concomitant biceps lesions. They may be associated with rotator cuff tears especially subscapularis tears or SLAP lesions. Also, isolated LHB pathologies may exist in the form of tendinitis, subluxation, partial, or complete tears [2–6].

Medical treatment with physiotherapy is the first option in treating mild forms of isolated biceps lesions. Surgical options vary widely and several techniques have been described in the literature either open or arthroscopic, tenotomy or tenodesis, and intra-articular or sub-pectoral [7, 8].

Several studies compared tenotomy and tenodesis. Although both procedures provide relief of pain and address the pathology in the LHB, tenotomy may result in some sort of cosmetic deformity (Popeye’s sign) and muscle cramps, especially in patients with adequate muscle bulk. Tenodesis, despite being more technically demanding, and requiring a longer time for rehabilitation and recovery, is the preferred surgical method for treating LHB pathologies [8–12].

Jamie L. Friedman et al. made a comparative study between tenotomy and tenodesis in active patients below 55 years old. Twenty-two cases underwent tenotomy versus 20 cases underwent tenodesis with a 3.3-year average follow-up. The tenodesis group had much better cosmetic results with less Popeye deformity compared to the tenotomy group. The time to fatigue, as well as the functional scores (DASH-VAS-ASES scores), was similar among both groups. However, the incidence of cramping was less in the tenodesis group (only one case) versus four cases in the tenotomy group [17].

Both tenodesis sites either supra-pectoral or sub-pectoral significantly improve the clinical outcomes and the studies that compared both methods found no significant differences between both techniques regarding most of the clinical outcomes or the post-operative complications [18].

In a comparative study made by Jun Tu et al. between **arthroscopic tenodesis** versus **open sub-pectoral tenodesis**, the differences were not significant regarding the demographic data and the operative time. The average time of hospital stay in the arthroscopic group was less than that in the open **sub-pectoral** group. On the other hand, the bicipital groove tenderness was higher in the arthroscopic than in the open group, especially in the early post-operative period. However, the final clinical and functional scores together with the post-operative complications showed no significant differences among both studied groups (*p* < 0.05) [19].

Multiple studies performed the intra-articular tenodesis with very promising results. Márcio Schiefer et al., in 2020, performed arthroscopic proximal biceps tenodesis with good clinical and isokinetic results when compared to the opposite healthy side. No cases required revision surgeries. Only one case (0.03%) reported mild sporadic pain in the bicipital groove [20]. Paul C. Brady et al. reported similar satisfactory results of intra-articular tenodesis in a large study performed on 1083 cases with 136 weeks of follow-up [21]. Brian M. Godshaw et al. in 2018 compared supra-pectoral versus intra-articular biceps tenodesis. They did not find a difference in the functional scores, although the intra-articular group had quicker improvement and larger scores but without statistical significance [22].

Several techniques were adopted for the fixation of the tenodesed tendon. Several studies used soft tissue fixation methods by suture fixation of the long head of biceps to the surrounding soft tissue structures. They have the advantage of being easy and rapid procedures and do not need an additional method of fixation [23].

F Franceschi et al. used a soft tissue procedure to tenodese the biceps with the Roman Bridge repair. They stated that despite being a relatively easy and rapid procedure, there have been some concerns regarding the biomechanical stability and the pull-out strength of the tenodesed tendon [24].

A Apivatgaroon et al. described an arthroscopic technique for fixation of the long head of biceps to the short head and coracoacromial ligament. Its main advantage is that it does not require additional fixation devices with added costs. However, it is more technically difficult, requires cautious haemostasis, and carries a risk of injury to the musculocutaneous nerve [25].

Tendon-to-bone fixation provides a more secure fixation than soft tissue fixation and is for sure more biomechanically stable. However, tendon-to-bone fixation methods will require a fixation device such as biodegradable screws or suture anchors [24].

Several studies used biodegradable screws, suture anchors, and other fixation devices [15, 16]. Cadaveric studies proved that biodegradable screws are biomechanically superior to suture anchors for biceps tenodesis [26].

The use of bio-screws requires bi-cortical drilling of the tenodesis site which makes the proximal humerus more liable to fractures as reported in some studies. This may be also attributed to the large drill holes created for the insertion of bio-screws. On the other hand, knotless anchors require only uni-cortical drilling that is performed by very small diameter drill bits [15, 16, 27].

In our prospective case series study, we introduced a new technique for fixation of the long head of biceps. It was done between the period of December 2019 and November 2022. Our study was done all through arthroscopically using the press-fit instruments.

There are several advantages to using press-fit bony plugs for biceps tenodesis. First of all, there are no implants used, and therefore, there are no additional costs for fixation. We only needed a trephine drill 8 mm in diameter which was used for every 15 cases (4000 EGP = 130 USD). So, each case costed 8.5 USD compared to 130 USD for the bio-screw cost and 322.5 USD (10,000 EGP) for the suture anchor cost. Second, the used method is a biological way with no implant-related problems regarding tunnel dilatation, risk of infection, or fractures. Third, the harvested bony plug is the same plug used for fixation in its original place abolishing the risk of tunnel mismatch. Fourth, the use of a bone plug for fixation will induce bone-to-bone union which is certainly faster to occur, and more secure than soft tissue procedures or tendon-to-bone healing. This will therefore allow rapid and safe rehabilitation programs. Fifth, it is done all through arthroscopically, with no open surgery. The fixation of the biceps tendon intra-articularly without entering the sub-acromial space and debridement of the bursa made the technique easier and more time-saving. Last but not least, being high in the groove avoids any neuro-vascular structure with no neuro-vascular complications.

In our study, the final average constant score was 94 post-operatively (range 85–98) (*p* value < 0.05). Also, the ASES and the VAS scores varied significantly from pre-operative scores of 43.3 and 6.5 points to 95 and 0.6 points respectively at the last follow-up with *p* value < 0.05. Although the ASES and constant scores improvement cannot be granted only to the improvement in the biceps function in cases of associated rotator cuff tears. However, some tests address only the biceps improvement such as the biceps strength when compared to the opposite healthy side, cosmetic deformity (Popeye’s sign), biceps cramping, and failure of the tenodesis. All these outcomes showed positive results in our study. Furthermore, the absence of bicipital groove-related pain, and tenderness at the final follow-up and the marked improvement in the VAS scores can be considered significant specific indicators of the improvement in the biceps tendon function.

Pascal Boileau et al. used bioabsorbable screws in the fixation of the tenodesed biceps tendons. There was an improvement in the absolute constant score at the last follow-up (79) points compared to (43) points in the pre-operative period. Fixation failure occurred in two cases early in the series, where tendon rupture occurred. The strength of the biceps muscle reached 90% of the opposite side with normal elbow extension and no neuro-vascular problems [13].

In the study made by Jun Tu et al., the mean VAS score in the open group improved from 5.02 to 0.95 at the final follow-up versus 4.92 pre-operatively and 1.18 at the final follow-up in the arthroscopic group. The constant score and the ASES at 12 months were 90.71 and 89.05 respectively in the open group versus 90.38 and 88.51 in the arthroscopic group. Stiffness occurred in three cases (5.5%) in the open group and 11 cases (17.7%) in the arthroscopic group [19].

In the comparative study made by Ji Soon Park et al., biceps tenodesis was done using both bioabsorbable screws and suture anchors. The patients were followed up for at least two years. The mean final post-operative VAS score was 0.6 in the bio-screw group and 0.8 in the anchor group. The final constant score was 94.9 in the bio-screw group and 93.8 in the suture anchor group. Anatomical failure of the tenodesis occurred in nine cases, seven in the bio-screw group (21%), and two cases in the suture anchor group (5.8%) [28].

When comparing our study to the previous studies, we found that we had comparable results that performed biceps tenodesis with variable techniques with fewer post-operative complications and with no external implant used (Table 2).

**Table 2:** Literature review

	Constant score	VAS score	ASES score	Complications
Our study	94	0.6	95	Reflex sympathetic dystrophy: 1 case (5%)
P Boileau et al	79	-	-	Failure: 2 cases (4.6%)
Jun Tu et al				Stiffness:
Open group: Arthroscopic group:	90.71 90.38	0.95 1.18	89.05 88.51	3 (5.5%) 11 (17.7%)
Ji Soon Park et al				Failure:
Bio-screws group: Anchors group:	94.9 93.8	0.6 0.8	- -	7 (21%) 2 (5.8%)

The limitations of our study include the relatively small number of cases with 2-year follow-up. A longer follow-up period with larger number of cases may be needed. Also, being a case series study not a comparative randomized study is another point to be taken into consideration.

## Conclusion

Our technique is safe and accessible with low economic demands. We recommend our technique to be used more often when addressing patients with long head of biceps pathologies.
